# Cardiovascular Disease in Children: The Future Is Now

**DOI:** 10.3390/children10050886

**Published:** 2023-05-15

**Authors:** Sebastiano A. G. Lava

**Affiliations:** 1 Pediatric Cardiology Unit, Department of Pediatrics, Centre Hospitalier Universitaire Vaudois and University of Lausanne, 1011 Lausanne, Switzerland; webmaster@sebastianolava.ch; 2 Heart Failure and Transplantation, Department of Paediatric Cardiology, Great Ormond Street Hospital, London WC1N 3JH, UK; 3 Clinical Pharmacology and Therapeutics Group, University College London, London WC1H 9JP, UK; 4 Division of Clinical Pharmacology and Toxicology, Institute of Pharmacological Sciences of Southern Switzerland, Ente Ospedaliero Cantonale, 6900 Lugano, Switzerland

Cardiovascular disease is a leading cause of morbidity and mortality worldwide [1], and an increasing body of evidence underscores the importance of foetal and paediatric programming for cardiovascular health and disease [1,2,3]. Fortunately, in recent years, the field of paediatric cardiology has witnessed an impressive increase in diagnostic and therapeutic possibilities.

A branch of paediatric cardiology deals with anatomical malformations and, with this respect, several advancements have emerged. First, following the major progresses in paediatric cardiac surgery in the second half of the last century [4], a plethora of interventional techniques have been established to efficiently address several structural problems without the need for open cardiac surgery and extracardiac circulation [4]. Second, recognising that perioperative survival is nowadays a quality criterium rather than an “achievement”, we now face the challenge of addressing middle- and long-term survival and morbidity, aiming at bringing this “congenital heart disease population” into their eighties in excellent health. This shifts the main focus of research from operative techniques to perioperative management and long-term continuous care, while requiring a thorough physiological, systemic paediatric and pharmacological understanding [5]. Third, novel echocardiographic techniques and progresses in cardiac MRI nowadays allow better assessment of systolic and diastolic function, synchrony, tissue characterisation and flow measurements, thus permitting detailed anatomical and functional assessments and, potentially, a more granular, personalised therapy.

A series of functional conditions is going to demand cardiological attention in the years to come. Genetic advances have opened new perspectives in the understanding of cardiomyopathies, rhythm disorders and blood vessel diseases. Pharmacological and interventional approaches, as well as pacemaker devices, are offering improved therapies to arrhythmias. Recent developments, including AB0-incompatible transplantation, donation after circulatory death, personalised immunosuppression and efforts in better understanding coronary allograft vasculopathy are advancing the field of heart transplantation. Recent, promising progresses in the pharmacotherapy of adult heart failure are going to be studied in children. In addition to strict cardiac conditions, such as myocarditis, pericarditis, cardiomyopathies or pulmonary hypertension, cardiovascular involvement in nephrological, oncological, haematological, neuromuscular, respiratory and metabolic (including diabetes mellitus) diseases is increasingly recognised [1,6]. The SARS-CoV-2 pandemic and the paediatric inflammatory multisystem syndrome are just recent examples of this “systemic nature” of contemporary paediatric cardiology. Furthermore, we are continuously raising our attention to cardiovascular health, from foetal life into adulthood [1,2]: perinatal conditioning, arterial hypertension, hypercholesterolemia, endothelial function and vascular health of solid-organ transplant recipients represent main challenges that need to be addressed [1,2,3,6].

In this Special Issue, dedicated to “Cardiovascular Disease in Children”, we are covering some of these aspects. From middle-term outcomes after anatomical surgery [7]; through application of percutaneous interventional techniques in rare paediatric syndromes [8]; vasovagal syncope [9]; the role of neonatal therapeutic hypothermia on QTc interval prolongation [10]; to echocardiography applications and perspectives [11,12]; inflammatory conditions such as Kawasaki disease in young infants and children [13] and multisystem inflammatory syndrome [12,14]; prevalence variations in arterial hypertension around the COVID-19 pandemic [15]; up to the exciting field of vascular health and cardiovascular biomarkers [16,17], we are inviting you on an inspiring journey.

## Data Availability

Not applicable.

## References

[1] Di Bernardo S.C., Lava S.A.G., Epure A.M., Younes S.E., Chiolero A., Sekarski N., Arhab A., Bovet P., Gilbert L., Gross J. (2022). Consequences of gestational diabetes mellitus on neonatal cardiovascular health: MySweetHeart Cohort study. Pediatr. Res..

[2] Barker D.J., Osmond C., Golding J., Kuh D., Wadsworth M.E. (1989). Growth in utero, blood pressure in childhood and adult life, and mortality from cardiovascular disease. Br. Med. J..

[3] Pool L.R., Aguayo L., Brzezinski M., Perak A.M., Davis M.M., Greenland P., Hou L., Marino B.S., Van Horn L., Wakschlag L. (2021). Childhood Risk Factors and Adulthood Cardiovascular Disease: A Systematic Review. J. Pediatr..

[4] Freedom R.M., Lock J., Bricker J.T. (2000). Pediatric cardiology and cardiovascular surgery: 1950–2000. Circulation.

[5] Lava S.A.G., Zollinger C., Chehade H., Schaffner D., Sekarski N., Di Bernardo S. (2023). Diuretics in pediatrics. Eur. J. Pediatr..

[6] De Ferranti S.D., Steinberger J., Ameduri R., Baker A., Gooding H., Kelly A.S., Mietus-Snyder M., Mitsnefes M.M., Peterson A.L., St-Pierre J. (2019). Cardiovascular Risk Reduction in High-Risk Pediatric Patients: A Scientific Statement from the American Heart Association. Circulation.

[7] Kwak J.H., Lee S.W., Cha H.R., Huh J., Kang I.-S., Jun T.-G., Yang J.-H., Han M.Y., Song J. (2022). Long-Term Observational Outcomes after Total Correction of Congenital Heart Disease in Korean Patients with Down Syndrome: A National Cohort Study. Children.

[8] De Simone L., Chiellino S., Spaziani G., Porcedda G., Calabri G.B., Berti S., Favilli S., Stefani L., Santoro G. (2023). Acute Coronary Syndrome Treated with Percutaneous Coronary Intervention in Hutchinson–Gilford Progeria. Children.

[9] Du X., Tao C., Wang Y., Sun Y., Zhang Q., Zhang C., Liu P., Wang Y., Liao Y., Du J. (2022). Twenty-Four-Hour Urinary Sodium Excretion Predicts Therapeutic Effectiveness of Oral Rehydration Saline in Pediatric Vasovagal Syncope. Children.

[10] Allegaert K., Salaets T., Ward R.M., Annaert P., Smits A. (2021). QTc Intervals Are Prolonged in Late Preterm and Term Neonates during Therapeutic Hypothermia but Normalize Afterwards. Children.

[11] Bressieux-Degueldre S., Fenton M., Dominguez T., Burch M. (2022). Exploring the Possible Impact of Echocardiographic Diastolic Function Parameters on Outcome in Paediatric Dilated Cardiomyopathy. Children.

[12] Di Nardo M., Franceschini A., Tissieres P., Chinali M. (2022). What Is New on Paediatric Echocardiography for the Diagnosis, Management and Follow-Up of the Multisystem Inflammatory Syndrome Associated with COVID-19?. Children.

[13] Roh D.E., Kwon J.E., Kim Y.H. (2022). Bacille Calmette-Guérin Site Reactivation of Kawasaki Disease in Infants under 3 Months of Age: Relation with Diagnosis and Prognosis. Children.

[14] Tunçer T., Varol F. (2022). Cardiovascular Outcomes in Children with Multisystem Inflammatory Syndrome Treated with Therapeutic Plasma Exchange. Children.

[15] Song K., Jung S.Y., Yang J., Lee H.S., Kim H.-S., Chae H.W. (2023). Change in Prevalence of Hypertension among Korean Children and Adolescents during the Coronavirus Disease 2019 (COVID-19) Outbreak: A Population-Based Study. Children.

[16] Senekovič Kojc T., Marčun Varda N. (2022). Novel Biomarkers of Heart Failure in Pediatrics. Children.

[17] Močnik M., Marčun Varda N. (2022). Current Knowledge of Selected Cardiovascular Biomarkers in Pediatrics: Kidney Injury Molecule-1, Salusin-α and -β, Uromodulin, and Adropin. Children.

